# Multistate Memristive Tantalum Oxide Devices for Ternary Arithmetic

**DOI:** 10.1038/srep36652

**Published:** 2016-11-11

**Authors:** Wonjoo Kim, Anupam Chattopadhyay, Anne Siemon, Eike Linn, Rainer Waser, Vikas Rana

**Affiliations:** 1 Peter Grünberg Institut 7, Forschungszentrum Jülich GmbH, 52425 Jülich, Germany; 2 Institut für Werkstoffe der Elektrotechnik II, RWTH Aachen University, 52074 Aachen, Germany; 3 School of Computer Science and Engineering, Nanyang Technological University, Singapore

## Abstract

Redox-based resistive switching random access memory (ReRAM) offers excellent properties to implement future non-volatile memory arrays. Recently, the capability of two-state ReRAMs to implement Boolean logic functionality gained wide interest. Here, we report on seven-states Tantalum Oxide Devices, which enable the realization of an intrinsic modular arithmetic using a ternary number system. Modular arithmetic, a fundamental system for operating on numbers within the limit of a modulus, is known to mathematicians since the days of Euclid and finds applications in diverse areas ranging from e-commerce to musical notations. We demonstrate that multistate devices not only reduce the storage area consumption drastically, but also enable novel in-memory operations, such as computing using high-radix number systems, which could not be implemented using two-state devices. The use of high radix number system reduces the computational complexity by reducing the number of needed digits. Thus the number of calculation operations in an addition and the number of logic devices can be reduced.

Redox-based resistive switching random access memories (ReRAMs) are considered as one of the most promising emerging non-volatile memory technologies^1^,^2^,^3^. The devices can be scaled down to 5 nm^4^,^5^, offer endurance up to 10^12^ cycles^6^, 10 years retention^7^ and fast read/write speed of below 200 ps^8^. The devices are switched to a low resistive state (LRS) for a positive SET voltage and switched to the high resistive state for a negative RESET voltage. Up to 8 multi-states have been shown^9^, allowing the storage of up to three binary digits in a single cell. Additionally ReRAM devices offer highly non-linear switching kinetics, i.e. the SET time depends exponentially on the pulse amplitude^10^. Due to abrupt switching events the common approach is to apply an external current compliance (CC) to enable multi-level resistance states^11^. The drawback of this approach is that the final resistance is defined by the CC, but not by the actual applied pulse amplitudes. However, a direct correlation between pulse height and final resistive state is feasible for a gradual RESET process, where a *V*_stop_ voltage defines the resistive state in advanced valence change mechanism (VCM) devices^12^,^13^. In this work, we use optimized Pt/W/TaO_x_/Pt ReRAM devices, offering highly reliable stop-voltage behavior and use the corresponding multi-level properties to implement modular arithmetic operations, as discussed in the result section.

Modular arithmetic finds usage in everyday applications, e.g., quantifying a specific clock-time, which wraps around after a fixed value is reached. A rigorous mathematical framework of modular arithmetic was developed by Carl Friedrich Gauss^14^ by defining a congruence relation between integers. Two integers, *a* and *b* are said to be congruent modulo *n*, when their difference (*a*-*b*) is divisible by *n*. In this case, *n* is known to be the modulus of this relation.


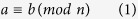


The properties of integer numbers for a specific modulus, spanning addition, subtraction and multiplication are written as following.

Given *a*_1_ ≡ *b*_1_(*mod n*) and *a*_2_ ≡ *b*_2_(*mod n*), we have


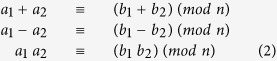


Apart from applications in mathematics, modular arithmetic plays a fundamental role in modern computer arithmetic. Here, a ring of integers modulo 2 is termed as a Boolean ring and every Boolean ring gives rise to Boolean algebra, where the ring multiplication is conjunction operator (∧) and the ring addition is exclusive disjunction operator (∨). Furthermore, the idea of secure and fault-tolerant data communication relies on the principles of public-key cryptography and error-correcting codes, respectively. Both of these fields require efficient implementations of modular arithmetic.

Modular arithmetic is also useful for reducing the complexity of standard arithmetic circuits^15^,^16^ and is essential for building the residue numeral systems (RNS). The RNS representation allows overflow-free addition, subtraction and multiplication, thereby enabling high degree of parallelism.

State-of-the-art modular arithmetic circuits in CMOS technology are implemented using two-state Boolean arithmetic operations, which follows directly from the two-level switching algebra introduced by Shannon^17^. Memristive devices were suggested to replace register files in conventional signed-digit adders^18^ or to be used in conjunction with complex quantization circuits^19^. The current paper reports the first implementation of modular arithmetic using multi-state ReRAM devices, which is fully crossbar array compatible in conjunction with a selector device. So far, most previous memristive circuit studies are based on over simplistic memristor models^20^, we hereby use real memristive devices fabricated in word structures to verify the proposed functionality. It should also be noted that we perceive no theoretical limit in scaling the number of states for memristive devices, thereby, opening a new research direction on multi-state storage and computing devices.

## Results

### Device properties

**Figure 1:**
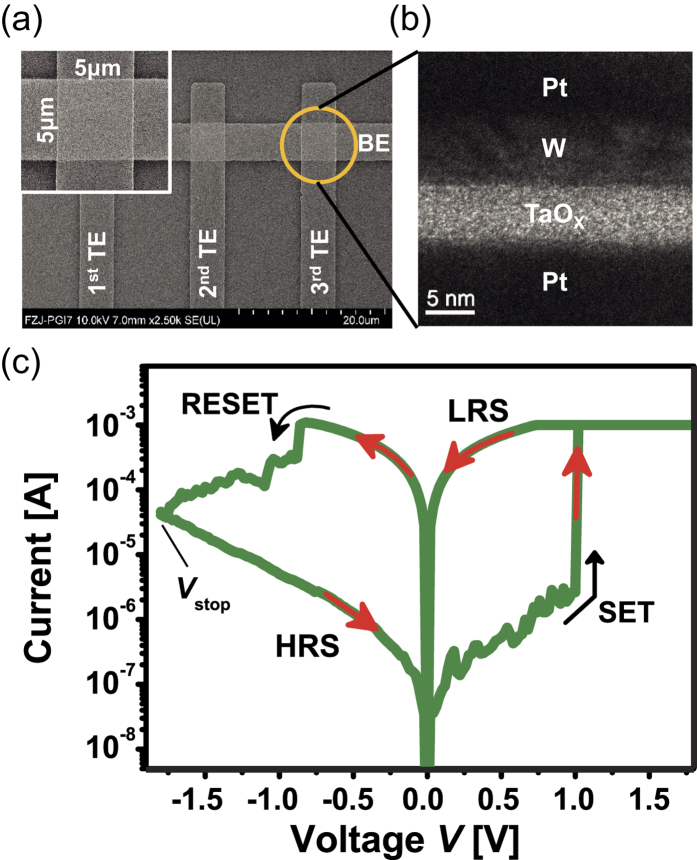
Resistive switching device structures. (**a**) Scanning electron microscopy image of 1 × 3 array with the inset showing 5 × 5 μm^2^ single device (**b**) Transmission electron microscopy image of single device cross-section, 7-nm-thick TaO_x_ switching layer and 13-nm-thick tungsten ohmic electrode. (**c**) Typical bipolar operation of SET-RESET switching in DC sweep mode for a single ReRAM (5 × 5 μm^2^) device within the 1 × 3 array.

In this work, 5 μm × 5 μm Pt/W/TaO_x_/Pt cross-point devices arranged in word structures (Fig. 1a) are used to realize the three Trit (**tr**inary dig**it**) modular addition. The device stack 25 nm Pt/ 13 nm W/7 nm TaO_x_/30 nm Pt is depicted in Fig 1b. The typical *I*-*V* characteristic of this device is shown in Fig. 1c. In supplementary S1, the *I*-*V* characteristic of an 80 nm × 80 nm cross-point device is also shown, highlighting the scaling potential of these devices. During RESET process, the maximum applied voltage |*V*_stop_| defines the final resistive state (1.8 V in Fig. 1c). This feature is also present in pulse mode, thus can be implemented in memory and logic operations for controlling the multi-level states.

**Figure 2:**
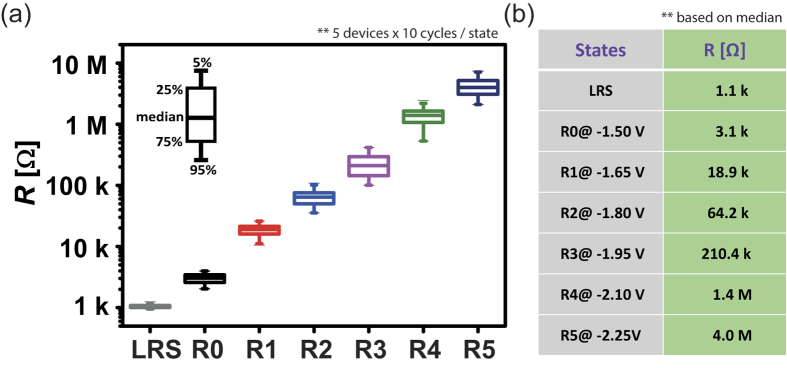
Resistance distribution. (**a**) Pulses of 200 ns and pulse height in the range of −1.5 V to −2.25 V (0.15 V steps) enable highly accurate resistive state control. (**b**) Mean values of the final resistance levels.

Figure 2a shows the cumulative probability of low resistance state (LRS) and six multi-level resistive states, which are obtained for 200 ns pulses in the range of *V*_stop_ = −1.50 V to −2.25 V. Each state is based on 5 devices with 10 cycles. The inset explains the statistical information of distribution. The tight distribution highlights the excellent switching properties of this device. In Fig. 2b, the mean value for each of the resistive state R0 to R5 is given.

**Figure 3:**
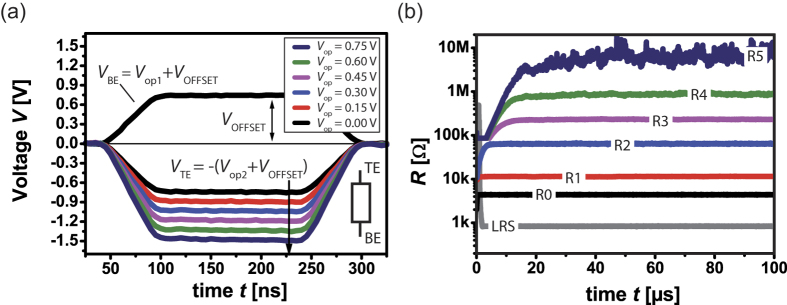
Basic logic functionality. (**a**) The logic operands *p* and *q* are applied to top (TE) and bottom (BE) electrode, respectively. An OFFSET voltage *V*_OFFSET_ is used to enable an equal stepping of operand voltages. In this example, *V*_op1_ = 0 V holds while *V*_op2_ is varied from 0 V to 0.75 V. (**b**) Depending on the pulse height, R0…R5 are written to the device. Here, a read-out voltage of 0.1 V was used to show the actual resistive states.

For the proposed arithmetic operation, the input operands are applied to the top (TE) and bottom (BE) electrode, respectively. To enable an equidistant voltage stepping, we use a predefined OFFSET voltage (*V*_OFFSET_) for each pulse. The operand voltages are *V*_op_ = 0.00 V to 0.75 V with increment of 0.15 V. The actual pulse applied at the bottom electrode is therefore *V*_BE_ = *V*_OFFSET_ + *V*_op1_ and *V*_TE_ = −(*V*_OFFSET_+*V*_op2_) for the top electrode. Thus, the overall potential difference is *V*_stop_ = *V*_TE_–*V*_BE_ = −(2 *V*_OFFSET_ + *V*_op1_ + *V*_op2_). Since the overall device voltage is always negative, a logic operation corresponds to a RESET pulse whose amplitude depends on the actual operands. To show the multi-level pulse operation mode, we set *V*_BE_ = 0.75 V and vary *V*_TE_ from −0.75 V to −1.50 V (Fig. 3a). The resulting resistances are depicted in Fig. 3b. Depending on the overall device voltage (*V*_stop_ = −1.50 V to −2.25 V) six different resistance states (R0, R1, R2, R3, R4 and R5) are easily accessible (Fig. 3b). Note that three resistive states would be sufficient to represent a ternary numeral system (Trit). This multi-level device property is used for the modular arithmetic operation. To enable highly reproducible RESET operation, we always apply a DC SET operation before each pulsed RESET operation. Note that also nanosecond pulsed SET operations are feasible, but not applied in this work. Details on the pulsed SET operation can be found in Supplementary S2.

### Developed Modular Arithmetic Working Principle

The new developed algorithm calculates the carries and sums directly in the ReRAM devices, which store the results until they are read out. Initially, all the devices in a wordline are initialized, i.e. written to the LRS. Starting from this state the sum bit of significance 0 (s_0_) can be directly calculated in the device of significance 0 while the other devices are calculating the first output carry c_1_. The actual sum or carry calculating devices are shifted for each significance one device to the left.

**Figure 4:**
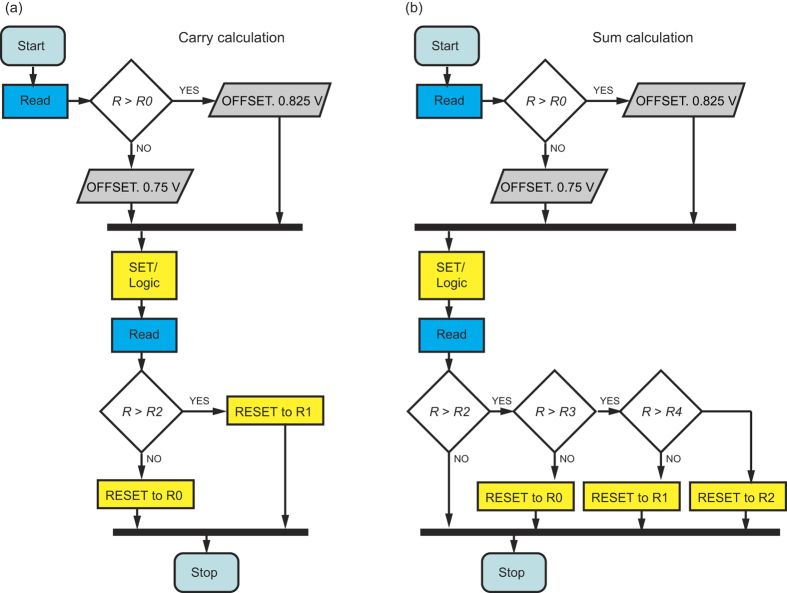
State machine. (**a**) Algorithm for carry calculation. First, the input carry is read and OFFSET is adjusted correspondingly. Next, the logic operation is conducted and high resistive states >R2 are mapped to R1 otherwise to R0. (**b**) Algorithm for sum calculation. The OFFSET evaluation and logic signal application is the same as for carry. The mapping is as follows: R3 → R0, R4 → R1 and R5 → R2. Start and stop of the algorithm are marked by light blue color, Read by blue color, and logic and RESET steps by yellow color.

In general, for the carry algorithm (Fig. 4a), first the device state of the actual device is read, to check whether the input carry *c*_in_ is 0 or 1. In case of 1, *V*_OFFSET_ is set to 0.875 V whereas in case of 0, the OFFSET remains *V*_OFFSET_ = 0.75 V. Next, the logic operation is conducted after a SET operation using the evaluated OFFSET. We apply *V*_TE_ = −(*V*_OFFSET_ + *V*_op1_) to the top electrode and *V*_BE_ = *V*_OFFSET_ + *V*_op2_ to the bottom electrode. Finally, the resistive state of the device is read and evaluated. To enable a proper modulus operation the ReRAM device has to provide 2*n* states for an *n*-ary number system. The background is that in a *n*-ary number system the operands at each specific significance are in the range of *0…n-1*, i.e., the sum of two operands is at most *2n-1*. Since, an input carry of 1 may also occur, the totally required number of states per device is *2n*. Thus, for a ternary number system six states (R0…R5) are required. If the state is R ≤ R2, the output carry *c*_out_ is 0 and R0 is written back. For R > R2, the device is written to R1, i.e. *c*_out_ = 1. Note that prior to the write back operation, the SET operation is conducted to enable a highly controlled R1 state. In supplementary S4, the required peripheral circuitry is depicted. Note that a certain minimum crossbar array size is required to justify the peripheral circuitry overhead. In this respect, a suitable selector device is a key component enabling large-scale ultra-dense multi-level ReRAM arrays.

Based on the input carry, the final sum can be calculated (Fig. 4b). As for the carry calculation, the required level of the *V*_OFFSET_ (either 0.75 V or 0.875 V) is first evaluated. Next, the operand voltages *V*_TE_ = −(*V*_OFFSET_ + *V*_op1_) and *V*_BE_ = *V*_OFFSET_ + *V*_op2_ are applied after the SET operation. Based on a final readout, the mapping R3 → R0, R4 → R1 and R5 → R2 has to be conducted to complete the modulo sum operation. The corresponding write back operation is done subsequently after the SET operation.

Since the signals which are applied to the TE are the same in both algorithm, these can be conducted in parallel on devices of different significance. Thus the cycle count can be kept low.

### Proof-of-concept

**Figure 5:**
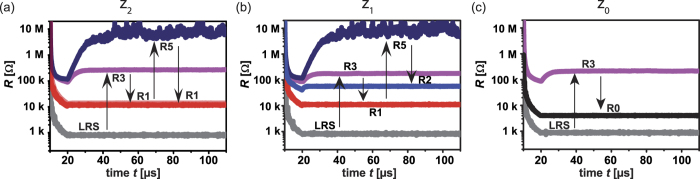
Proof of concept measurement. This example is for p = 21 (2·3 + 1 · 1 = 7) and q = 22 (2·3 + 2 · 1 = 8). (a) The sequence of resistive states in ReRAM device z_2_, (b) ReRAM device z_1_ and (c) ReRAM device z_0_. The final values are: z_2_ = R1, z_1_ = R2 and z_0_ = R0. This corresponds to 1·9 + 2 · 3 + 0·1 = 15. Numbers and arrows indicate the state changes. In between SET-steps are not shown. Full data for this example can be found in supplementary S4–S6.

For the proof-of-concept measurement, a two Trit modular addition is selected, adding the ternary numbers **p** = p_1_p_0_ and **q** = q_1_q_0_. Since the sum output **z** = z_2_z_1_z_0_ needs three Trit digits, three ReRAM devices are required for this operation and initialized to LRS firstly. The addition is performed in a word-line structure (cf. Fig. 1a). For the exemplary addition, operand 1 is **p** = 21 (=7) and operand 2 is **q** = 22 (=8). Note that input 0 corresponds to *V*_op_ = 0.00 V, input 1 corresponds to *V*_op_ = 0.15 V and input 2 corresponds to *V*_op_ = 0.30 V, using the earlier described incremental stepping of 0.15 V. In Fig. 5a–c, the sequentially obtained resistive states are shown. The arrows mark the order of steps without showing the in between SET-steps.

The algorithm described in Fig. 4 realizes the following mathematical modulo sum operation:

In device z_0,_ the sum operation is conducted directly:

- z_0_ = (1 + 2) rem 3 = 0 (s_0_).

Note that the function ‘rem’ returns the remainder. Starting from LRS, the device is reset (p_0_ = 1 = > *V*_TE_ = −0.9 V and q_0_ = 2 = >*V*_BE_ = 1.05 V, i.e. −1.95 V). According to Fig. 2b, this voltage leads to state R3, as can be also seen in Fig. 5c directly. According to the sum algorithm (Fig. 4b), R3 is finally mapped to R0, see Fig. 5c.

In device z_1_, first the carry operation is conducted:

- z_1_ = (1 + 2) div 3 = 1 (c_1_ = 1).

The function ‘div’ returns the floor quotient. Starting from LRS, the device is toggled to R3 state by applying p_0_ and q_0_ to calculate the carry c_1_. According to the carry algorithm (Fig. 4a), R3 is then mapped to R1, see Fig. 5b.

Next, the second cell sum Trit is obtained by the following operation:

- z_1_ = (2 + 2 + c_1_) rem 3 = 2 (s_1_).

Since c_1_ = 1 holds the *V*_OFFSET_ = 0.875 V is applied, and the accessed state is R5. According to the sum algorithm (Fig. 4b), R5 is then mapped to R2, see Fig. 5b.

For cell z_2_ (Fig. 5a), again the carry operation is conducted first:

- z_2_ = (1+2) div 3 = 1 (c_1_ = 1).

Starting from LRS, R1 state is accessed via R3.

Since we consider a two Trit addition, the final sum bit equals the carry c_2_:

- z_2_ = (2 + 2 + c_1_) div 3 = 1 (c_2_ = s_2_ = 1).

According to the carry algorithm (Fig. 4a), R5 is then mapped to R1, see Fig. 5a.

The final sum is stored directly in memory:

- Sum **z** = z_2_ z_1_ z_0_ = 120 (=15).

**Figure 6:**
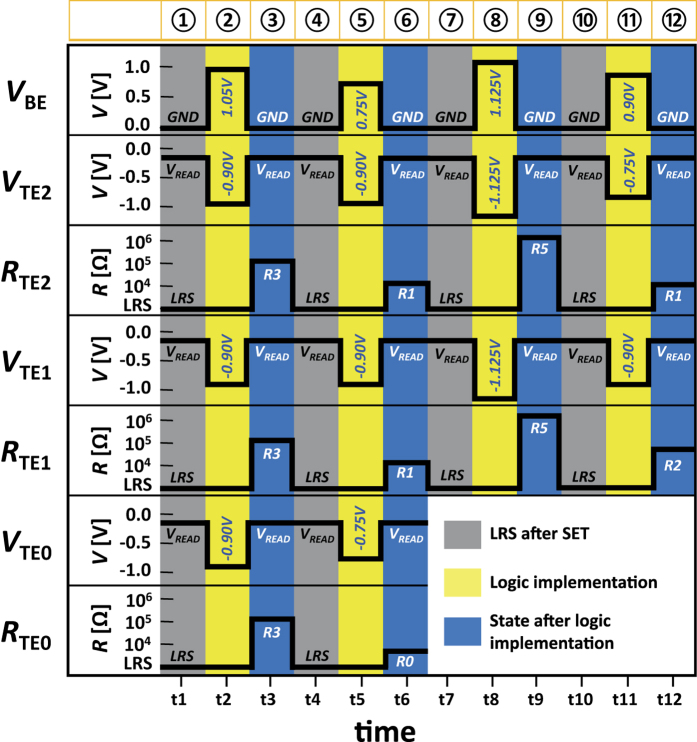
Schematics of operation. Schematics of operation for applied voltages (*V*_TE_, *V*_BE_) are shown. The gray shows the LRS after SET. The yellow depicts the logic implementations and the blue shows the corresponding states after the logic implementation. Step 1 is the LRS after initialization and Step 2 is logic implementation. Step 3 is the resistance states based on Step 2. Step 4 is the LRS after SET and Step 5 implements the RESET for the modulo operation. Step 6 is the corresponding resistance states and Step 7 is the LRS after SET. Step 8 is the logic implementation with adjusted OFFSET and Step 9 is the corresponding resistance states. Step 10 is the LRS after SET. Step 11 is the logic implementation and Step 12 is the corresponding resistance states.

**Figure 7:**
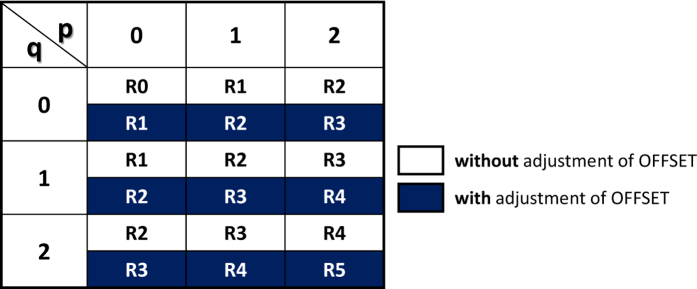
Truth table for state definition. Truth table of p and q to realize each corresponding state with and without the OFFSET adjustment.

In Fig. 6 the schematics of applied operation voltages and corresponding states are depicted. The first line shows the voltages at the common bottom electrode (BE) acting as a wordline (WL). The second, fourth and sixth lines show the voltages applied to the three separate top electrodes (*V*_TE2_, *V*_TE1_, *V*_TE0_) acting as bitlines (BL) while the third, fifth and seventh lines represent the resistance states (*R*_TE2_, *R*_TE1_, *R*_TE0_) at each BL. The three background colors are used. The gray shows the LRS after SET. The yellow depicts the logic implementations and the blue shows the corresponding states after the logic implementation. Overall twelve steps are presented. Step 1–2 show the initialized LRS and logic implementation. Step 3–4 depict the corresponding resistance states with the the LRS after SET. Step 5–6 show implementations and the corresponding states. The state is set to LRS in Step 7. The logic is implemented with adjusted OFFSEET in Step 8 and the corresponding states are shown in Step 9. The LRS after SET is shown in Step 10. Step 11–12 show the logic implementations and the corresponding resistance states. The states (*R*_TE2_, *R*_TE1_, *R*_TE0_) shown in Step 12 and Step 6 depict the final sum stored in memory (Sum **z** = z_2_ z_1_ z_0_ = 120). The more details of overall steps are given in Supplementary S4-S6. A truth table for the overall state definition (R0 – R5) is shown in the Fig. 7. Each combination of p (TE) and q (BE) sets the corresponding state with and without the adjustment of OFFSET.

## Discussion

We have demonstrated a ternary number system implementation, using multi-states tantalum oxide devices in word structures. Depending on the available number of resistive states, higher order number systems can also be implemented in the same way. For *n*-ary systems, we would need 2*n* resistive states, hence further progress in ReRAM memory technology will directly enable arithmetic operations using higher radix number systems.

On the other hand, the choice of radix for a number representation can be motivated from the perspective of underlying implementation as well as the analysis of radix economy. A quantifiable measure of radix economy proposed in ref. 21 is as following:


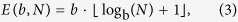


where, *b* is the radix and *N* is the number to be represented. This metric yields *E* as the most economical real-valued radix. It also turns out that the radix value of 3 (ternary) is more economical compared to binary. We argue that the above measure does not take the growth of the implementation media into account. For several device technologies, the area requirement grows linearly with the radix size and make the radix implementation very tough. However, it is not true for multistate memristive devices such as the Pt/W/TaO_x_/Pt ReRAM, since the implementable radix size depends on the number of resistance states. Considering, a *k*-state device can be realized at the same cost of a two-state device, a more appropriate metric would be


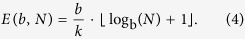


Compared to binary arithmetic, an *n*-ary number representation reduces the space complexity in a logarithmic ratio. Given comparable performance for the base devices, the gain in arithmetic circuits, such as, integer addition is also expected to be in logarithmic scale. However, the actual gain will be somehow smaller due to need for better sense amplifiers and more control circuitry.

The presented approach is not limited to a specific multistate ReRAM device, but would work for any memristive device offering multiple resistance levels induced by different stop voltages *V*_stop_. The proposed algorithm could further be simplified by avoiding in between SET operations, however this requires ultra-low variance ReRAM devices. For the considered ReRAM device only RESET pulses were allowed as logic inputs. Appropriate SET pulses enabling step-by-step decrease of the resistance could be used to implement also subtraction within the same device similar to the here shown additional operation.

The presented approach is compatible to the passive crossbar array configuration, by integrating a selector device to each TaO_x_ junction. The implementation of the arithmetic functionality within the resistive memory device using the available multi-resistance levels is a highly attractive option for future functionality enhanced hybrid CMOS/ReRAM chips. This approach enables a reduction of cycle count compared to Boolean logic based ReRAM approaches^22^,^23^,^24^,^25^. For example, a recently proposed cipher application could be decisively improved using multi-level ReRAMs^26^, enabling efficient in-hardware encryption and decryption for future smart devices. The energy per operation depends on the device properties, namely switching voltage, multi-level resistive states and inherent switching speed and control circuit properties such as the applied pulse width. Since the pulse width (t) that will be used in real application is much shorter than 200 ns (used in study), the final power consumption will be reduced further. In summary, low-variance multi-level ReRAM could play a key role for implementation of public-key cryptography and error-correcting codes in smart devices.

## Conclusion

Pt/W/TaO_x_/Pt devices enable highly reliable multi states, which can be accessed reproducibly by pulses of specific height, starting from a defined LRS. By using word and bit lines as inputs for pulses, the resistive multi-levels can be used to store and calculate in-memory logic operations. To avoid an overflow in individual devices, a modulus arithmetic is implemented, assuring the device to be always in a valid 0, 1, 2 (Trit) state. By using a ternary number system, the amount of devices and cycles can be reduced significantly. In contrast to two-state devices, multistate devices provide better radix economy with the option for further scaling. Therefore, establishing multi-state ReRAM for non-volatile memory opens the door to novel storage and in-memory computer arithmetic options.

## Methods

### Device Fabrication

Devices are fabricated for 1 × 3 array with crossbar structure based on 5 × 5 μm^2^ single cell size. Three top electrodes shares single bottom electrode. For the bottom electrode (BE), 5 nm titanium (Ti) and 30 nm platinum (Pt) layers are deposited by sputtering on top of thermally grown SiO_2_ layer (430 nm). A photolithography and dry etch processes are applied to pattern the bottom electrode. And then 7.0 nm-thick TaO_x_ is deposited by reactive sputtering under process gas mixture of Ar (23%) and oxygen (7%) with the RF power of 116W at the chamber pressure of 2.3 × 10^−2^ mbar. Without breaking the vacuum, 13 nm tungsten (W) ohmic electrode, and 25 nm platinum (Pt) are deposited consecutively. And then top electrode (TE) is patterned with a photolithography and a reactive ion etch. A scanning electron microscopy of 1 × 3 crossbar array with 5 × 5 μm^2^ size cell is shown in Fig. 1a and its corresponding cross-sectional structure with tunneling electron microscopy is shown in Fig. 1b.

### Measurement Set-up

Initially the resistive switching devices remain in high resistance state (HRS) and we need to apply irreversible forming process in order to activate the devices before repetitive switching cycles are possible. Detailed information is given in supplementary section. During the forming process, the resistance state of device changes from HRS to LRS. In order to change the resistance state from LRS to HRS, a ‘RESET’ process is required. In reverse way, a ‘SET’ process converts the HRS to LRS. The DC operation of a single device also known as current-voltage (*I-V*) sweep is shown in Fig. 1. The voltage sweep is applied only to the top electrode (TE) while the bottom electrode is grounded. In order to achieve better control on the resistance state of device with single pulse operation, the ‘SET’ process is based on DC operation. However, the other operations such as ‘RESET’ and ‘read’ use AC pulse operation. For the RESET operation, 200 ns pulse width based on full width half maximum (FWHM) is applied for switching operations with 40 ns rising/falling times. The read operation at 0.1 V uses 120 μs long pulse in order to verify the each resistance value more accurately, especially HRS values. By applying a 0.15 V stepping, seven resistive levels can be distinguished in the considered devices. Depending on the actual devices even more multi-level states are feasible in ReRAM devices. However, due to variability, e.g., induced by random telegraph noise^27^, the maximum number of properly accessible multi-levels is limited.

## Additional Information

**How to cite this article**: Kim, W. *et al*. Multistate Memristive Tantalum Oxide Devices for Ternary Arithmetic. *Sci. Rep*. **6**, 36652; doi: 10.1038/srep36652 (2016).

**Publisher’s note:** Springer Nature remains neutral with regard to jurisdictional claims in published maps and institutional affiliations.

## Supplementary Material

Supplementary Information
