# Supplementary material for: Multistate Memristive Tantalum Oxide Devices for Ternary Arithmetic
Source: Sci Rep. 2016 Nov 11;6:36652. doi: 10.1038/srep36652 (PMC5105152; doi:10.1038/srep36652)
Supplement: Supplementary Information [file srep36652-s1.pdf]

SUPPLEMENTARY INFORMATION

Multistate Memristive Tantalum Oxide Devices for Ternary Arithmetic

Wonjoo Kim[1], Anupam Chattopadhyay[3], Anne Siemon[2], Eike. Linn[2], Rainer Waser[1,2], Vikas Rana[1]

[1] Peter Grünberg Institut 7, Forschungszentrum Jülich GmbH, 52425 Jülich, Germany

[2] Institut für Werkstoffe der Elektrotechnik II, RWTH Aachen University, 52074 Aachen, Germany

[3] School of Computer Engineering, Nanyang Technological University, Singapore

Corresponding author: Dr. Vikas Rana (v.rana@fz-juelich.de)

Supplementary

S1 Comparison Nano devices – Micro devices

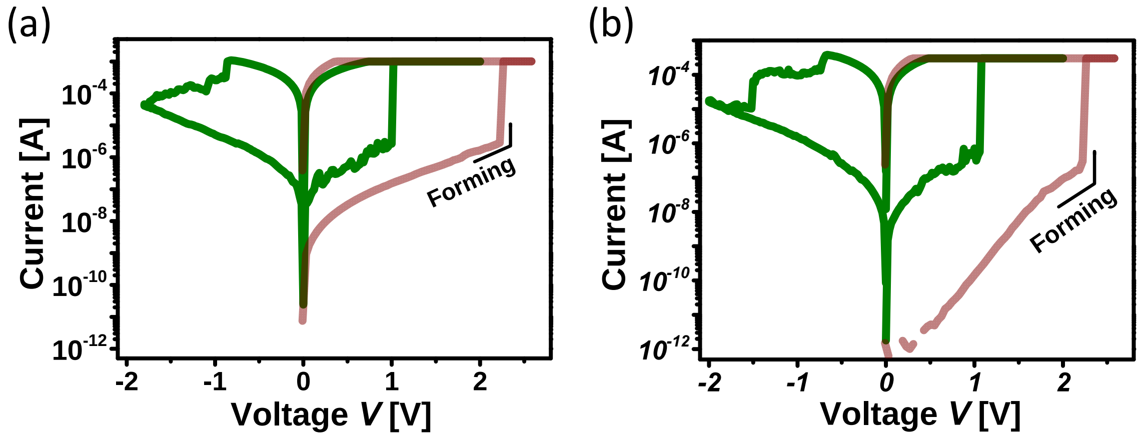

Figure S1 Typical *I-V* characteristics of (a) 5 µm x 5 µm micro device and (b) 80 nm x 80 nm nano device. Both devices have the layer stacks of 25 nm Pt / 13 nm W /7 nm TaO_x / 30 nm Pt. Both devices require a forming process as an initialization before RESET and SET processes. Their *I-V*-characteristic are very similar to each other.

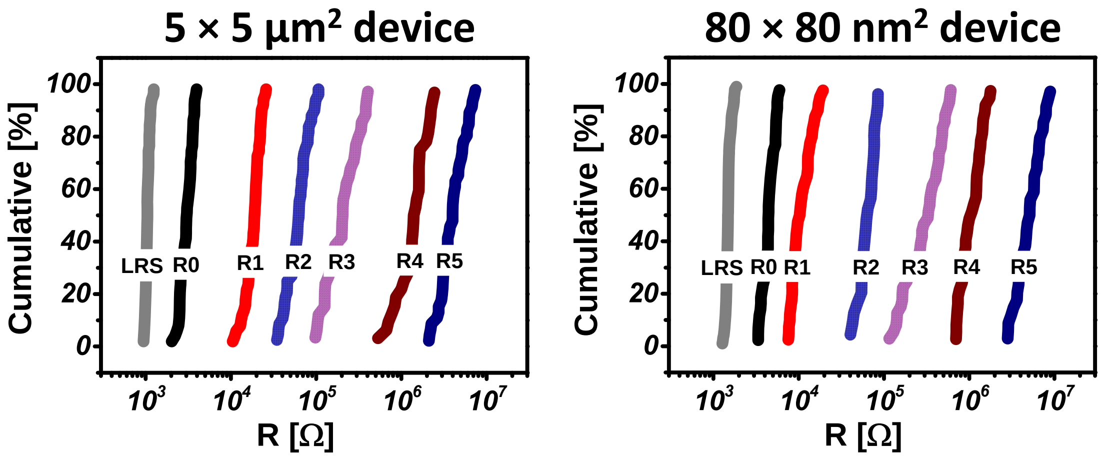

Figure S2 Comparison of resistance distribution for micro- and nano-meter ReRAM devices. The nanometer device also offers the tight distribution of the resistive states.

S2 Pulsed SET operation

<table>
<tr><th>after RESET (@200ns)</th><th>SET (1.5V@200ns)</th><th>after SET</th></tr>
<tr><td>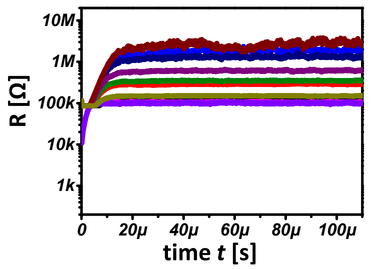
</td><td>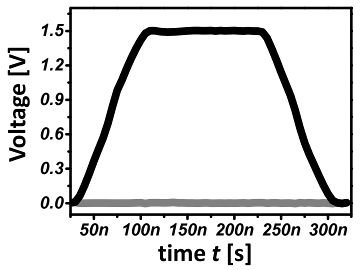
</td><td>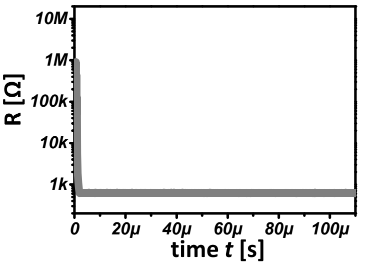
</td></tr>
<tr><td>READ: 0.1V@120µs</td><td></td><td>READ: 0.1V@120µs</td></tr>
</table>

Figure S3 Feasibility of pulsed SET operation. After the RESET operation, the ReRAM device is in a multi-level state (left). A SET pulse of 1.5 V (middle) sets the device to the LRS (right).

S3 Full data of the proof-of-concept measurements

z_0 device in 1 × 3 array

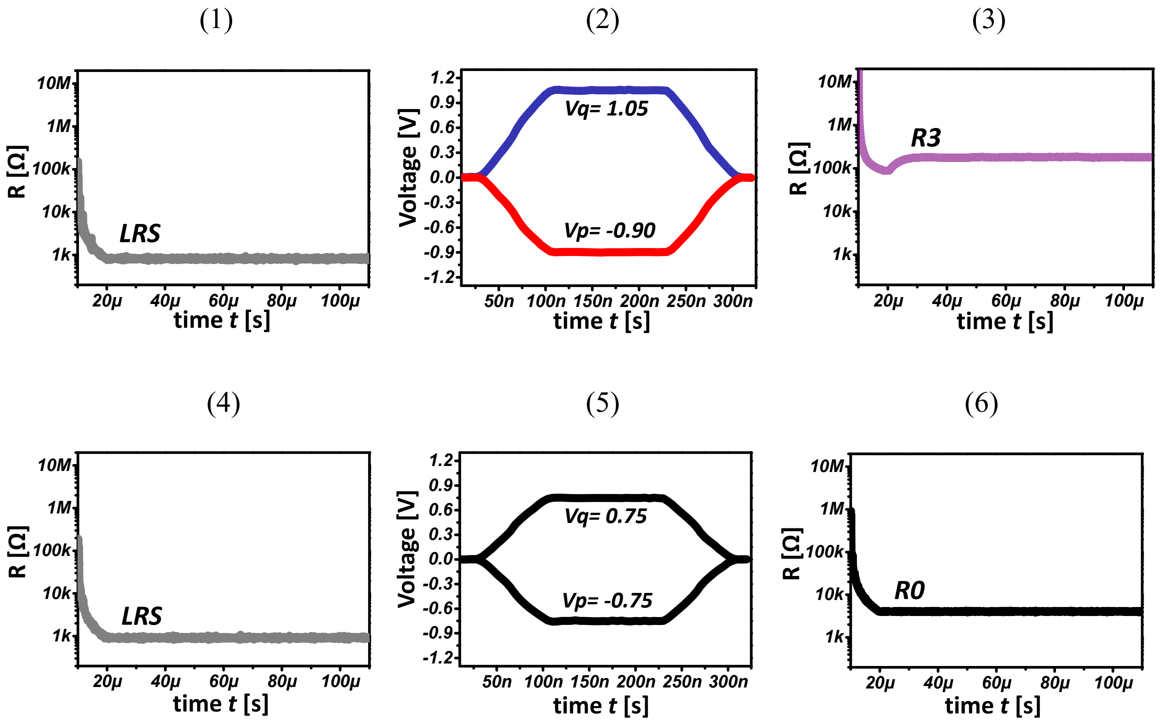

Figure S4. Sum calculation in z_0 step-by-step. (1) Initialization: SET to LRS. (2) Sum calculation. (3) State is R3 → Mapping to R0 required. (4) LRS after SET. (5) Write to R0. (6) Final state R0. Color code for voltages: see Fig. 3a. Color code for resistances: see Fig. 3b.

z_1 Device in 1 x 3 array

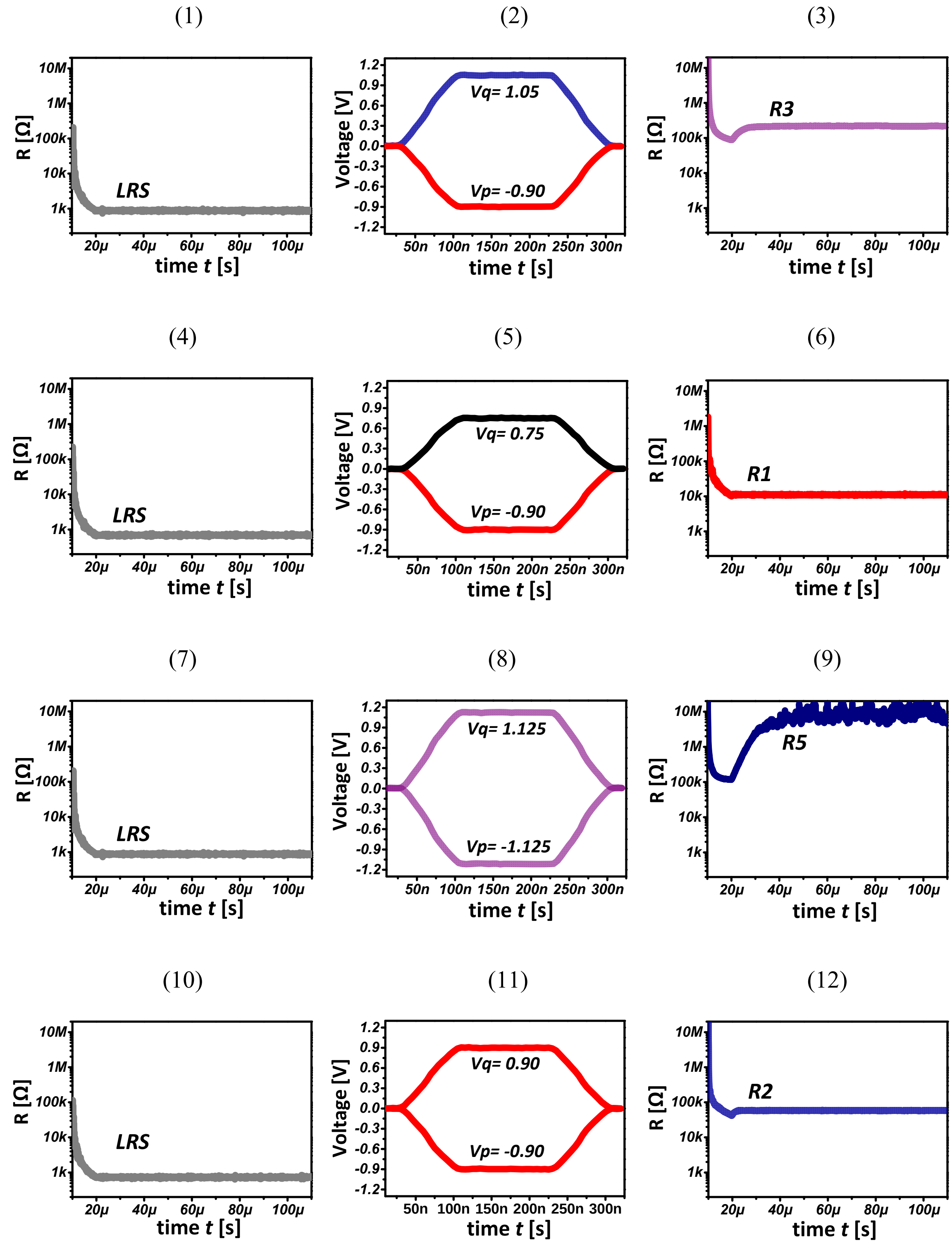

Figure S5. Sum calculation in z_1 step-by-step. (1) Initialization: SET to LRS. (2) Carry calculation. (3) State is R3 → Mapping to R1 required. (4) LRS after SET. (5) Write to R1. (6) State is R1. (7) LRS after SET. (8) Sum calculation. (9) State is R5 → Mapping to R2 required. (10) LRS after SET. (11) Write R2. (12) Final state R2.

Color code for voltages: see Fig. 3a. Color code for resistances: see Fig. 3b.

z_2 Device in 1 x 3 array

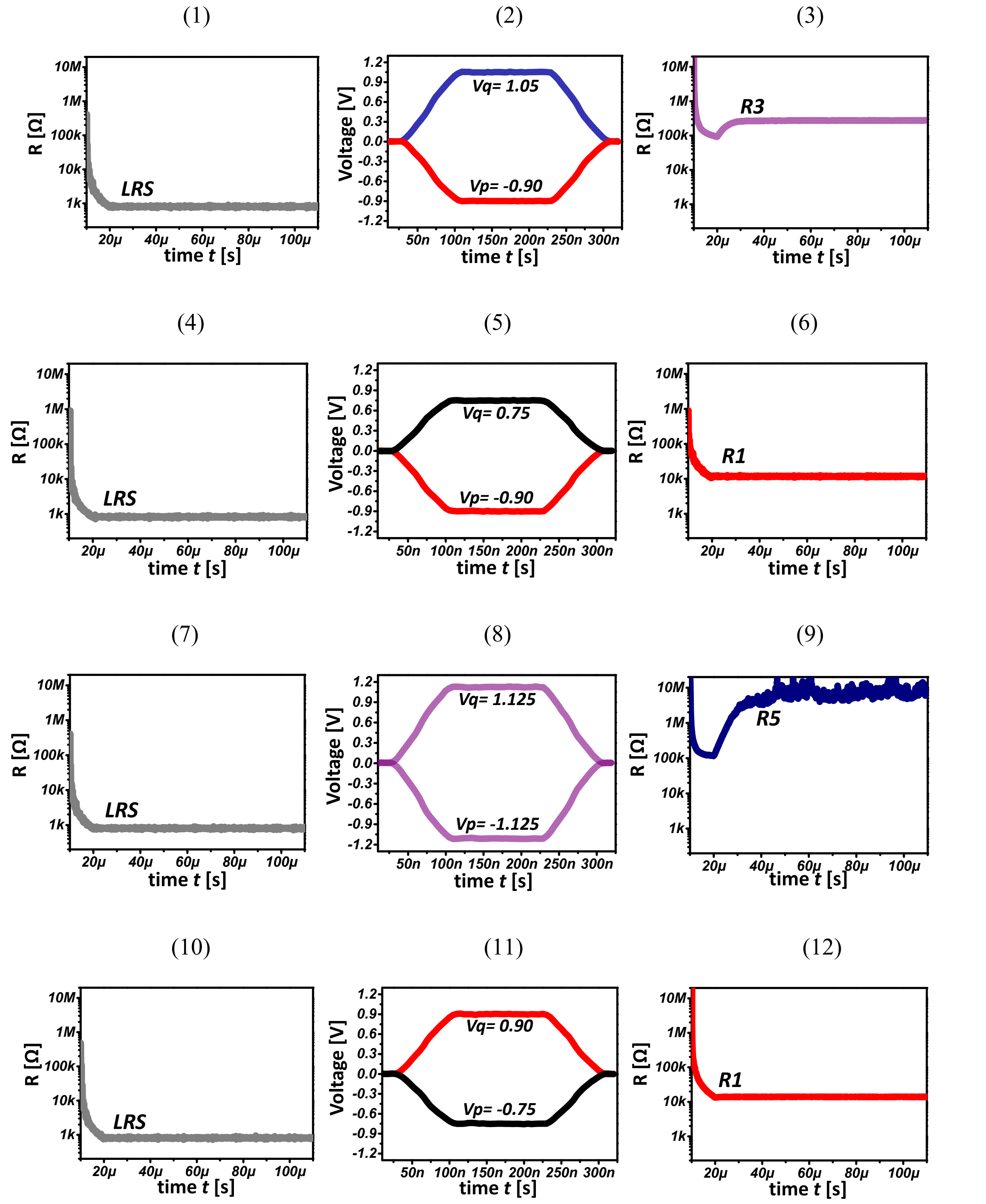

Figure S6. Sum calculation in z_2 step-by-step. (1) Initialization: SET to LRS. (2) Carry calculation. (3) State is R3 → Mapping to R1 required. (4) LRS after SET. (5) Write to R1. (6) State is R1. (7) LRS after SET. (8) Carry calculation. (9) State is R5 → Mapping to R1 required. (10) LRS after SET. (11) Write R1. (12) Final state R1.

Color code for voltages: see Fig. 3a. Color code for resistances: see Fig. 3b.

S4 Peripheral Circuitry

To enable modular arithmetic, one additional control block is required implementing the state machine.

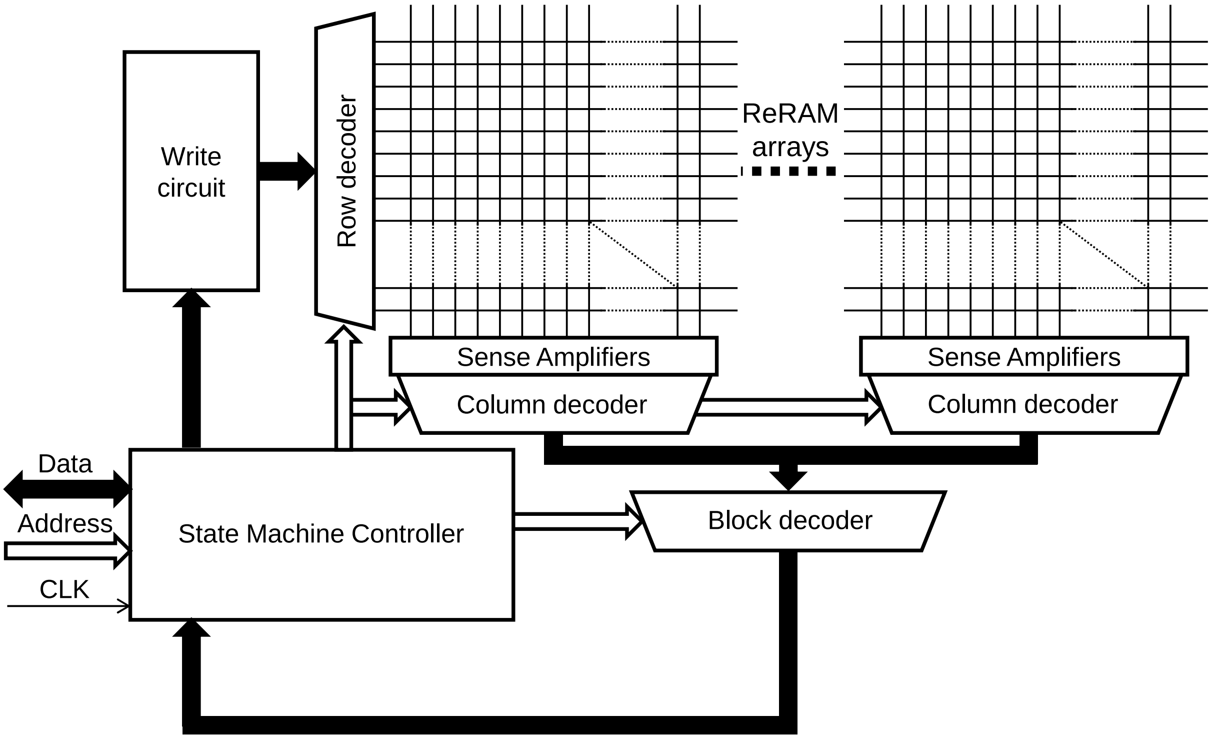

Figure S7. Peripheral Circuitry. The crossbar ReRAM memory arrays are controlled by a write circuitry, sense amplifier, row, and column and block decoders. Additionally, a state machine controller is required to support the in-memory operation. This circuitry implements the state machines drawn in Fig. 4, especially the OFFSET, logic and SET/RESET control.
